# Health system response to preventing mother-to-child transmission of HIV policy changes in Zambia: a health system dynamics analysis of primary health care facilities

**DOI:** 10.1080/16549716.2022.2126269

**Published:** 2022-10-14

**Authors:** Jonathan Mwanza, Mary Kawonga, Andrew Kumwenda, Glenda E. Gray, Wilbroad Mutale, Tanya Doherty

**Affiliations:** a School of Public Health, University of the Witwatersrand, Johannesburg, South Africa; b Department of Community Health, Charlotte Maxeke Johannesburg Academic Hospital Johannesburg, Johannesburg, South Africa; c Department of Obstetrics and Gynaecology, School of Medicine, University of Zambia, Lusaka, Zambia; d Office of the President, South Africa Medical Research Council, Cape Town, South Africa; e School of Public Health, University of Zambia, Lusaka, Zambia; f Health Systems Research Unit, South Africa Medical Research Council, Cape Town, South Africa

**Keywords:** Health system, prevention of mother-to-child transmission of HIV (PMTCT), primary health care, ART, eMTCT, zambia

## Abstract

**Background:**

Zambia is focusing on attaining HIV epidemic control by 2021, including eliminating Mother to Child Transmission (eMTCT) of HIV. However, there is little evidence to understand frontline healthcare workers’ experience with the policy changes and the readiness of different health system elements to contribute to this goal.

**Objective:**

To understand frontline healthcare workers’ experience of preventing mother-to-child transmission (PMTCT) of human immunodeficiency (HIV) policy changes and to explore the health system readiness to respond to rapid changes in PMTCT policy by using the health system dynamic framework.

**Method:**

We conducted a qualitative study in which 35 frontline healthcare workers were selected and interviewed using a snowball sampling technique. All transcripts were analysed through thematic content analysis and deductive coding. Themes were derived and presented according to the health system dynamics framework.

**Results:**

Among the ten elements of the health system dynamics framework, service delivery, context, and resources (i.e. infrastructure and supplies, knowledge and information, human resource, and finance) were critical in implementing the continuously evolving PMTCT policies. Furthermore, due to the fragmented primary health care platform in Zambia, non-governmental organisations (NGOs) were instrumental in ensuring that the PMTCT programme met the demand and requirements of the general population. Frontline healthcare workers who participated in the study described inequity in access to ART services due to the service delivery model employed in the selected study sites.

**Conclusion:**

The study highlights challenges when policies are implemented without consideration for the readiness, context, and capacity in which the policy is implemented. We offer lessons that can inform implementation of universal health coverage of antiretroviral therapy (ART), a strategy many countries have adopted, despite weak health systems.

## Background

Over the past decade, tremendous gains have been made in preventing mother-to-child transmission (MTCT) of HIV worldwide [1]. Expanded access to and uptake of antiretroviral therapy (ART) during pregnancy has helped to drive significant global reductions in MTCT of HIV [2,3]. Currently, there is robust evidence indicating that well-functioning health systems with forward-thinking PMTCT policies and programmes could almost eliminate MTCT under ideal conditions, and at a programmatic level <5% transmission could be possible in resource-limited settings [2,4,5]. As of 2019, in sub-Saharan Africa, ART coverage was estimated to be 84% among pregnant women living with HIV [6]. As a result, an estimated 420,000–580,000 infants have died due to MTCT of HIV and almost all paediatric HIV infections occur in sub-Saharan Africa, where the prevalence of HIV infection in women of childbearing age can reach 35% or more [7].

In Zambia, MTCT is one of the drivers of the HIV epidemic with 10% of all new HIV infections, and 90% of infections in children attributed to MTCT [8]. HIV prevalence in Zambia among adults aged 15–59 years is 11.5% (12.1% among females and 10.9% among males) corresponding to approximately 1200,000 people living with HIV (PLHIV) within this age group [9,10]. Approximately 66,000 children aged 0–14 years are living with HIV and 6,000 new infections were reported in 2019 within this age group [11].

In response to the HIV epidemic, the Zambia national policy for PMTCT 2007–2009 was adopted based on WHO PMTCT Option A/B [8]. It stipulated that women eligible for lifelong Antiretroviral Therapy (ART) options A/B would be those with absolute CD4 count ≤350cells/mm^3^ [12]. Within the same period, the Zambia Ministry of Health (MOH) integrated PMTCT interventions into Maternal and Child Health (MCH) services to help reduce MTCT of HIV [13] and to decrease both maternal and child mortality [8]. To further reduce an estimated 10,000 new HIV infections among children 0–14 years as a result of MTCT of HIV [14], Zambia adopted Option B+ as a new strategy within the PMTCT programme in 2013. In that same year, the programme recommended that all infants born to HIV-positive mothers should have a virological antigen test for HIV within the first 6 weeks and at 6 months of life [8]. Previously, no virological testing of infants was undertaken. This was followed by the Zambia National policy for PMTCT of 2016 [15], 2018 [16], and 2020 [17]. Following each policy change, new recommendations were introduced, including: new drugs for both the mother and child, the timing of virological antigen tests for HIV-exposed children (HEC) changed from 6 weeks, 6 months, and 9 months, to birth, 6 weeks, 6 months, 9 months, 12 months and 18 months, with clinical follow-up until the end of breastfeeding [17].

Achieving the MTCT of HIV goal of eliminating new HIV infections by 2021 required identifying obstacles and highlighting the changes required to facilitate the successful adoption and implementation of PMTCT policies in primary health care study settings. In this study, the focus of analysis is the health system readiness (HSR) to absorb rapid PMTCT policy changes and frontline healthcare workers’ experiences of these policy shifts. These frontline healthcare workers are considered street-level bureaucrats because they fulfil the main characteristics defined by Lipsky [18]; they interact directly with the citizens in the PMTCT policy implementation and its re-design. Readiness refers to the extent to which an organisation is willing and able to implement a particular innovation [19,20]. It is considered a necessary precursor to a successful organisation or policy change; thus, it is often embedded with more extensive programme planning and implementation [21,22]. HSR focuses on the preparedness of health care systems and institutions to accept the change due to new policies or to integrate new services [23]. To address the limited literature on these issues, our research sought to apply the health system dynamics framework (citation) to understand frontline healthcare workers’ experience of Zambia’s PMTCT policy changes from 2007 to 2020 and to explore the health system’s readiness to respond to rapid changes in PMTCT policy. The framework additionally assisted in outlining PMTCT policy gaps; and conditions needed for its successful implementation in the context of rapid policy changes.

## Method

### Study setting

The study was undertaken in Kitwe and Lufwanyama districts of the Copperbelt Province, Zambia. This province was selected because in 2020 it had the second-highest population in the country estimated at 2,669,635 [24], and had the third-highest HIV prevalence at 14.2% among the population [25]. The study was conducted in an urban study district (Kitwe), with the highest population in the province at 762,950 [26], and a rural district (Lufwanyama), with the highest population among the rural districts at 105,156 [26]. This allowed documentation of experiences in both urban and rural settings. Table 1 sets out the study districts selected health demographic characteristics for the year 2020 [27].

### Study design

This qualitative study employed in-depth interviews (IDIs) to collect data from frontline healthcare workers in primary care health facilities and hospitals in each study district.

### Participants

A total of 40 frontline healthcare workers were purposively selected, including health managers at the district and hospitals (Kitwe Central Hospital and Lufwanyama District Hospital) as well as frontline healthcare workers involved in operational planning and implementation within PHC facilities **(Table 2)**. The interviewees were purposively identified based on their experience with and involvement in PMTCT programme planning, decision-making and implementation in their respective health facilities and institutions. Frontline healthcare workers were recruited into the study only if they had work experience in the public healthcare sector (Ministry of Health) for five years or more, experience in PMTCT and consented to participate. Priority was given to primary health care facilities with a high volume of PMTCT clients and classified as delivery centres.

**Table 1.** Selected health demographic characteristics of Kitwe and Lufwanyama districts for the year 2020 [27]. District.

	Kitwe	Lufwanyama
Infant DNA PCR positivity	1%	0.05%
ART uptake	98%	54%
Antenatal Care (ANC) HIV testing	85%	83%
ANC HIV positivity	4%	3%
HIV prevalence	13.8%	3.12%
Expected pregnancies	24111	3657
Expected deliveries	23677	3591
Women of childbearing age	45769	26623
Under ones	22997	3488
Under Fives (U5)	109846	16304

Source: District Health Information System Lufwanyama and Kitwe 2020.

**Table 2.** Study participant characteristics.

Location	Number of PHC facilities	n	Type of participant
Kitwe District	15	3 17	program managers/coordinators (nurses) frontline healthcare workers (nurses)
Lufwanyama District	11	2 8	program managers. Frontline healthcare workers (nurses)
Hospital	1	1 4	Paediatrician Paediatric nurse
Total	27	35	

Key informants from health facilities were identified using the snowballing sampling technique that involved asking each informant after the interview if they knew anyone else who would have information related to the study. The key informants identified were then contacted either physically at their health facilities or electronically through email or phone calls and asked to participate in the study. If they agreed, an appointment was set. Written consent for all interviews and audio recordings was obtained for all interviews conducted.

Only 35 (88%) identified participants were available and recruited for this study. The five who chose not to participate cited different reasons, including urgent matters to attend to within their facilities.

### Data collection

Data collection for this study was carried out between February 2019 and July 2020 using in-depth interviews. The interview guides were pilot tested in non-study sites to ensure the questions were correctly understood and to estimate the time to complete the interviews. All interviews were conducted face-to-face in English at participants’ workplaces by the researcher and four assistant researchers, lasted 25–30 minutes, and were audio-recorded with the consent of the participants.

### Data analysis

Data analysis proceeded simultaneously with data collection, and emerging findings informed deeper inquiries in subsequent interviews. The audio-recorded interviews were transcribed verbatim by the researcher and two research assistants, and a thorough accuracy check was done on two randomly selected transcripts to validate the accuracy of the transcription process. The electronic transcripts were then loaded into Nvivo 12 Pro qualitative data software [17], for data management. Analysis was based on a combination of thematic content analysis and deductive coding.

**Figure 1.**
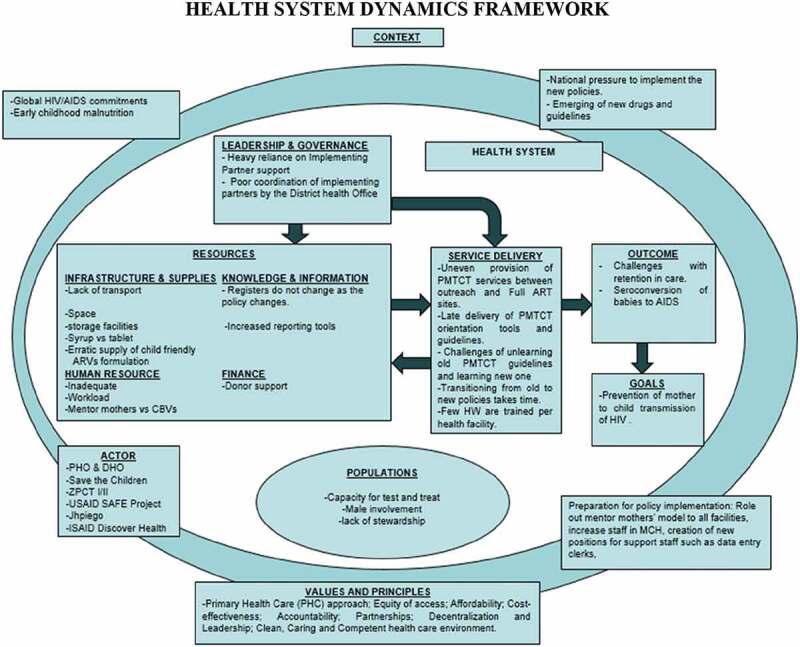
The health system dynamics framework adapted from Van Olmen [28] populated with the findings from this study.

The results were reported in a narrative form and presented according to the health system dynamic framework ten elements: context, population, leadership, governance, resources (infrastructure and supplies, human resources, knowledge, and information, finances), service delivery, outcome, and goals [28,29] as illustrated in Figure 1. The premise of this framework is that the different elements interact with each other and impact programme goals and outcomes. This framework strengthens the understanding of how the different elements interact with each other and how they impact the ultimate goals and outcomes of the PMTCT programme during implementation.

## Results

### Context

Some key informants believed that shifts in PMTCT policy and programme implementation in Zambia were influenced by increased cases of early childhood malnutrition and high mortality of women due to the HIV epidemic. In the respondents’ views, a key contextual reason for policy shifts to lifelong ART was the unintended rise in early childhood malnutrition due to the previous policy of giving ART only in pregnancy.

‘*Most of the mothers died of HIV as a result. We noticed that children were getting malnourished because the majority of the mothers could not afford appropriate complementary foods. Since mothers were not on lifelong treatment, their health deteriorated rapidly and a majority of them died,’ (Health worker in charge, PHC Facility, Kitwe district).*

Participants appreciated the policy change because they could see benefits such as the improved health status of the mothers.

‘*We appreciate the policy directive to put HIV-positive breastfeeding women on lifelong antiretrovirals (ARVs) as this has helped a lot of mothers live healthily and breastfeed their infants,’(Maternal & Child Health Coordinator, PHC Facility, Kitwe district).*

However, some participants were quick to mention the critical challenges the programme is currently experiencing.

*‘Although a lot of children are coming out positive at 18 months due to non-adherence to treatment by mothers and reduced clinical visits which we are not doing as often because of staff shortages,’(Maternal & Child Health Coordinator, PHC Facility, Kitwe District).*

### Population

All nurses interviewed acknowledged the benefits of the PMTCT programme, reporting that they had progressively embraced new initiatives and expressed their full support for the PMTCT programme. *‘During our PMTCT clinic at our health facility, we usually give priority to women who come with their partners. The reason we do that is to rebrand our services to look more male-friendly and encourage male involvement,’ (MCH Coordinator, PHC Facility, Lufwanyama District).*

Active involvement of family support structures among pregnant women living with HIV was considered important, especially in the urban district. Participants felt that antenatal and postnatal health services have their challenges. They emphasised the need for more efforts to bring in family support to improve PMTCT uptake. *‘There is so much stigma attached to HIV and as a result, new HIV-positive mothers are reluctant to come to the facilities. The introduction of mentor mothers (MM) who are HIV-infected mothers trained and employed by NGOs to work in selected model sites to give one-on-one support to HIV infected pregnant/postpartum women has helped, especially with the uptake of ARVs and disclosure of their HIV status to their partners,’ (Midwife, PHC Facility, Kitwe District).*

### Leadership and governance

Participants indicated the critical role of Implementing Partners (IPs) in providing resources towards supporting the district action plans, including funding to certain health programme areas, incentives to frontline healthcare workers and community-based volunteers, purchasing of equipment, provision of transport, medical supplies, and building infrastructure. This improved the readiness of the district health system. *‘The IP donation of a utility vehicle to district XX was really important because, at the time of the donation, the district did not have a reliable vehicle to conduct PMTCT mentorship programmes and referral of patients especially pregnant women between different levels of health care as well as making it easy for the transportation of personnel, goods, and services,’ (PMTCT focal Point Person, PHC facility, Lufwanyama District).*

The reliance on IPs for resource provision was particularly highlighted by a respondent from a rural facility. *‘The presence of this IP made a lot of difference because they managed to build us a fully furnished maternity wing and mothers shelter in selected facilities that are hard to reach. They have been in this district for the past ten years and right now there is no IP in the district. I hope your research will bring this to the attention of the authority and how that has affected us as a rural district,’ (PMTCT focal Point Person, PHC facility, Lufwanyama District).*

### Resource

#### Infrastructure and supplies

The change to Universal Test and Treat (UTT) and lifelong ART in 2013 necessitated frontline healthcare workers initiate all HIV-positive mothers and for postnatal mothers, their infants on ART within a week of first contact, irrespective of CD4 count. However, participants across all levels mentioned challenges with adhering to the PMTCT policy recommendations citing shortages of friendly paediatric ART formulations and the heavy reliance of outreach sites on static sites for certain services. *‘The biggest challenge we have in the PMTCT programme is adhering to guidelines due to out of stock of friendly paediatric ART formulation prescribed in the guidelines. As a health worker, I do not recommend ARV tablets for infants because it is challenging to reconstitute the solution for the babies and there are no specific containers for such,’ (Paediatric Nurse, Hospital)*.

*‘The paediatric ART formulation Kaletra (lopinavir/ritonavir) is thermosensitive and we are worried about storage of the drug in a home setting,’ (Paediatric Specialist, Hospital).*

Respondents also spoke of inadequate physical infrastructure space and that certain facilities were better equipped than others: ‘*As a result, UTT in the district is uneven and there are certain times when HIV-positive clients do not visit the outreach sites especially if we are not going there which means we will make them seem to be defaulting when in actual sense it is us who have not just visited those sites,’ (Midwife, PHC Facility, Kitwe District).*

#### Human resources

HIV-related health care demands increased as new policies were implemented at health facilities, especially among pregnant women attending antenatal care (ANC), putting workers under increasing pressure. Frontline healthcare workers who had been involved in service delivery described how challenging it had been to implement various new PMTCT policies because of insufficient health workforce. *‘In hospitals, there is adequate human resource compared to primary healthcare (PHC) facilities. There are still plenty of PHCs manned by one personnel who is expected to manage the outpatient department (OPD) functions, conduct special clinics like under 5, PMTCT, and outreach activities so we have a serious shortage of staff in certain facilities in the district,’ (District ART and PMTCT Coordinator, Kitwe District*).

However, in trying to redress human resource shortage challenges, non-governmental organisations (NGOs) especially those supported by United States Agency for International Development (USAID), recruited MMs to improve the human resource situation at the health facility level.

*‘Mentor mothers perform functions such as linkage to ART care, adherence, and retention in HIV care, perform tracing for women who miss clinic visits and provide health education on PMTCT,’ (Midwife, PHC Facility, Kitwe District).*

Some nurses emphasised the need for the government to adopt the MMs model as a national initiative. As it has the potential to improve the PMTCT programme, especially in human resource-constrained settings. They further added; *‘MM, perform certain functions which frontline healthcare workers could not perform due to other main responsibilities which they have at health facilities.’(General Nurse, Kitwe District).*

#### Knowledge and information

The PMTCT policy shift from Option A to UTT brought about new clinical and administrative responsibilities, such as introducing new registers. However, these registers were not provided to all health facilities and even when they were they required frontline healthcare workers’ orientation. Respondents described the situation as tedious as they had to fill in more than five PMTCT registers before submitting to the next reporting level. ‘*The following registers were being used at the primary health facilities that frontline healthcare workers filled in routinely; ANC, family planning (FP), integrated family planning, Under Five, mother-baby follow up, Dry Blood Spot (DBS), daily activity registers- ANC, FP, postnatal and viral load. Unfortunately, these registers do not correspond with the practices in the new policies,’ (General Nurse, PHC Facility, Lufwanyama District).*

Frequent changes in the PMTCT and ART guidelines also presented challenges in ensuring that all frontline healthcare workers understood and captured the correct data as required by the updated guidelines. *‘We have a lot of gaps in terms of data capturing. A frontline healthcare worker who is not oriented or trained in PMTCT will have difficulties adjusting to the new reporting format,’ (MCH Coordinator, PHC Facility, Lufwanyama District).*

### Finances

The financial sustainability of the PMTCT programme was a recurring theme. Several respondents described the inconsistent funding for PMTCT and its vulnerability to other competing needs within the districts. ‘*Some of the activities which get affected due to inadequate funding are mentorships because, when we have new guidelines, we need one-to-one mentorship with facilities staff; transport challenges when the need to visit health facilities to ensure adherence and implementation of new guidelines arises, as well as conducting data verification exercises,’ (MCH Coordinator, PHC Facility, Lufwanyama District).*

Despite numerous financial challenges, respondents acknowledged the important role of non-governmental stakeholders in providing external funding to the programme. *‘NGOs are the most active in implementing new policies. They fund the orientation meetings and provide the necessary logistics such as per diem or transport refund for frontline healthcare workers. However, I have a feeling that districts or facilities that are not supported by NGOs face different situations in their implementation of PMTCT or new guidelines,’ (Nurse, PHC Facility, Kitwe District).*

Service delivery (outreach sites and established ART sites)

The major challenge within the service delivery platform was related to outreach sites; which were not suitably prepared to provide comprehensive PMTCT services.

*‘Outreach sites are primary health care facilities that are not ART accredited by the Health Professional Council of Zambia (HPCZ) and do not meet certain standards to provide comprehensive PMTCT services such as limited laboratory and diagnostic services, human resources, and lack of physical infrastructure,’ (ART and Tuberculosis coordinator, Kitwe District).*

However, respondents highlighted the presence of NGO’s stepping in to support the service sites to deliver PMTCT services as per the new policy.

*‘NGOs have lessened the burden as they are helping to upgrade most of the facilities by expanding the physical infrastructure space, attaching skilled human resources, and supporting PMTCT activities,’ (PMTCT Coordinator, Lufwanyama District).*

### Outcome and goals

The major goals of the PMTCT programme are: the prevention of HIV infection among women of childbearing age, preventing unintended pregnancies among women living with HIV, preventing HIV transmission from a woman living with HIV to her infant, and treatment, care, and support to women living with HIV, their children, and families. Nurse participants wholeheartedly described that despite the positive outcome of the PMTCT policy changes, retention in care and seroconversion remained a challenge: *‘I have observed that the PMTCT yield has improved, recently. Most of the mothers have accepted the programme. I feel elimination is possible,’ (Nurse, PHC Facility, Kitwe District)*.

*‘We are putting a lot of emphasis on PMTCT and even mothers are not refusing because they have seen the importance of starting the medication to prevent MTCT. However, we are recording a lot of loss to follow-up exposed babies from our program starting at 9 months and when we see them at later months they are testing positive for HIV,’ (Nurse, PHC Facility, Kitwe District).*

## Discussion

This qualitative study contributes to the limited body of knowledge on health systems’ readiness to respond to rapid change in PMTCT policy. Our study explored the perspectives of health care workers and programme coordinators using the health system dynamics framework [28]. The study findings reveal that elements of the health system relating to service delivery, context, and resources (infrastructure and supplies, knowledge and information, human resources, and finance) are critical challenges in implementation of the PMTCT programme and undermine goals for achieving the elimination of MTCT of HIV in Zambia. These findings are similar to other studies conducted elsewhere on health system strengthening in Zambia [30] and South Africa [31].

Our findings illuminate the complex, social and adaptive nature of the primary health care level and demonstrate the usefulness of the health system dynamic framework to explain how and why primary health care services perform or underperform as they implement policies made at the central level without considering requirements at the district and facility level of health care delivery.

Our findings have shown that the absorption of new PMTCT policies at primary healthcare facilities is influenced by health system elements such as service delivery. Under this element, frontline healthcare workers held concerns of uneven provision of PMTCT services between the different health facilities. - Specifically, static ART sites/Established ART Clinics were accredited health facilities offering comprehensive PMTCT services that were able to conduct laboratory investigations on an HIV/AIDS positive client while Outreach Sites were not accredited but depended on static sites to offer ART services. Most of the health facilities in our two study districts were outreach sites and because of this, they had limited physical infrastructure, particularly if they did not have IPs support.

One of the major weaknesses of sub-Saharan African (SSA) health systems is inadequate human resources [32]. Africa is said to have less than one health worker per 1000 population [33]. Respondents in our study affirmed the insufficient number of frontline healthcare workers, which they described as leading to high workloads, worsened by unequal distribution of health professionals between hard-to-reach and not-hard-to-reach facilities, in rural and urban districts. To address human resource shortages in the health sector, studies have suggested several initiatives such as using mentor mothers [34,35]. In our study, this initiative was more noticeable in facilities supported by external implementing partners such as non-governmental organisations (NGOs) supported by USAID. These supported facilities had a community-based volunteer (CBV) cadre in the form of mentor mothers who supported frontline healthcare workers in the PMTCT programme. Mentor mothers and similar lay HIV frontline healthcare workers are persons living with HIV without a specific qualification [36,37]. Despite not being formally adopted at the national level, mentor mothers work in health facilities, in clients’ homes, and in the larger community and ultimately act as a link between health facilities and communities [38]. Our study highlights that the mentor mother initiative improved health system readiness to implement the PMTCT programme, by strengthening human resource and service delivery requirements.

Our findings support prior studies that have reported that the existence of CBVs such as MMs has eased the workload at health facilities and during special clinics such as the ‘Umoyo clinic’ [36] (translated as ‘clinic of life’) [39], a local mother-infant pair (MIP) initiative where all HIV positive breastfeeding women, their children, and expectant mothers come to the facility to receive integrated HIV and routine post-natal care services on a day separate from HIV-negative mothers [39]. Although results from studies done elsewhere indicate that the Umoyo programme did not have a positive impact on the retention of HIV-exposed infants within PMTCT care [39], respondents in our study indicated that this initiative has helped to increase social support among HIV-positive mothers and reduced stigma and thus improved retention of mothers and their infants in the facilities where this initiative had been implemented.

ART adherence among children is a crucial part of managing human-immunodeficiency virus (HIV) infection and extending the life and health of infected children [40]. Important causes of poor adherence are formulation- and regimen-specific properties, including poor palatability, enormous pill burden, short dosing intervals, and the complex storage and transportation of drugs [41]. This study highlighted that one of the critical challenges with health system preparedness to deliver the PMTCT programme and achieve good outcomes was the erratic supply of child-friendly ARV formulations, particularly drugs in syrup form. Respondents described that paediatric ARVs formulation in solid forms was challenging to administer to infants, especially in cases where only adult tablets were available. Syrups allow for proper dosing in young children [39,40]. Similarly, studies in many developing settings have found that the entire stock-out of a drug or stock-outs of a particular paediatric formulation required regimen alteration and led to confusion [39–41]. Consequently, inconsistencies in the availability of paediatric formulation or stock-outs of nevirapine (NVP) will leave caregivers with no option but to crush the available adult pills to reconstitute a solution for the infants, which does not guarantee an appropriate dosage. Also, crushing pills or opening capsules can reduce the bioavailability of the ART because the entire contents may not be administered, thus significantly reducing the targeted therapeutic exposure [42,43]. This can potentially reduce viral suppression and promote viral or drug resistance.

Another critical finding from this study was the issue related to palatability and refrigeration [43,44]. WHO recommends lopinavir/ritonavir as the first-line ART to initiate in children [45]. Protease Inhibitors such as Kaletra (Lopinavir/Ritonavir) are liquid forms that ease administration in infants who cannot swallow tablets [46]. However, respondents described these Protease Inhibitors (PI), such as (Kaletra) lopinavir/ritonavir, as thermosensitive and bitter [47]. Many studies described this bitter taste to have been found to impact adherence in infants and that Kaletra (Lopinavir/ritonavir) refrigeration requirements can become a problem in resource-limited settings where electricity is not always available in patients’ homes [46].

Our study is limited in that only the views and experiences of midwives or facility maternal and child health coordinators (MCH) are presented. Our approach was to gather information on their views and experience with PMTCT programme implementation. Obtaining views on the issue from other frontline healthcare workers within the facility may have yielded additional perspectives on PMTCT implementation. Additionally, exploring the community-level experience could potentially fill gaps in understanding their views and experiences of the PMTCT programme implementation; this was not addressed in this paper.

## Conclusion

The findings from this study exposed challenges faced by health care workers in the context of rapid policy change. The study also confirms the relevance of the health system dynamics framework, an analytic approach to understanding the critical elements of the health system when implementing different PMTCT policies over time.

As is the case for many LMICs, Zambia should consider some of the issues raised by frontline healthcare workers as they continue to implement PMTCT and other programmes with rapid policy guideline changes, such as tuberculosis. Continuous investment and stakeholder involvement in the programme must be sustained to strengthen the health system elements. The study constitutes an essential contribution to health policy and systems research, generating findings that may act as a building block for programme improvement and performance in LMICs.

## Paper Context

Zambia is striving to attain HIV epidemic control, including eliminating Mother to Child Transmission (MTCT) of HIV. However, little is known about the readiness of different health system elements to contribute to this goal. The study highlights challenges when policies are implemented without consideration for health system readiness, context, and capacity. We offer lessons for universal health coverage of antiretroviral therapy, a strategy many countries have adopted, despite weak health systems.
